# Extraneural recurrence of an intracranial nongerminomatous germ cell tumor to cervical lymph nodes in a pediatric patient: Case report

**DOI:** 10.1002/cnr2.1586

**Published:** 2021-11-18

**Authors:** Jackson Howell, Christopher Dandoy, Jordan M. Wright, Lionel Chow, Ayman El‐Sheikh, Mukund Dole, Ralph E. Vatner, Kambiz Kamian

**Affiliations:** ^1^ Department of Radiation Oncology University of Cincinnati College of Medicine Cincinnati Ohio USA; ^2^ Cancer and Blood Diseases Institute Cincinnati Children's Hospital Medical Center Cincinnati Ohio USA; ^3^ Department of Hematology/Oncology Dayton Children's Hospital Dayton Ohio USA; ^4^ Department of Neurosurgery Dayton Children's Hospital Dayton Ohio USA

**Keywords:** germ cell tumor, salvage therapy, high‐dose chemotherapy

## Abstract

**Background:**

Intracranial germ cell tumors (GCTs) comprise 3%–5% of pediatric primary central nervous system (CNS) tumors in Western countries. Though they are related in embryonic origin to gonadal GCTs, which are considered highly treatable with cisplatin‐based chemotherapy regimens, intracranial GCTs vary in malignant potential and sensitivity to radiation and chemotherapy, generally carrying a worse prognosis. Metastases of intracranial GCTs outside of the CNS are rare, indicate a poor prognosis, and their salvage treatment is not well established.

**Case:**

A 15‐year‐old boy presented with bifocal (suprasellar and pineal) intracranial nongerminomatous germ cell tumors of mixed origin. The tumors were treated to full response with a multimodal approach of neoadjuvant chemotherapy, surgical resection, and adjuvant craniospinal proton radiation. Nine months following treatment completion, the patient presented with an enlarged cervical lymph node determined on excisional biopsy to be a recurrence of pure germinoma from the primary tumors. Salvage treatment involved high‐dose chemotherapy and autologous stem cell transplantation; however, the patient denied further treatment prior to planned focal radiotherapy. Thirty months post‐treatment, the patient is well with no evidence of recurrence.

**Conclusion:**

This case demonstrated the successful salvage treatment of an extraneural recurrence of an intracranial GCT using surgical resection and a high‐dose chemotherapy and autologous stem‐cell transplantation regimen, highlighting the unique factors which led to the selection of this regimen.

## 1 INTRODUCTION

Intracranial germ cell tumors (GCTs) comprise 3%–5% of pediatric primary central nervous system (CNS) tumors in Western countries, with a higher incidence in East Asia.^1^ These tumors derive from the inappropriate migration of embryonic germ cells and are, thus, related in character to gonadal germ cell tumors (GGCTs).^2^ There are multiple histologically derived classifications of GCTs, and malignant potential and radiosensitivity vary by class.^3^ Though GGCTs are considered highly treatable with cisplatin‐based chemotherapy, intracranial GCTs have a more diverse response, and their prognosis is typically less favorable.^2^, ^4^

We report a case of a pediatric patient who experienced an isolated extraneural recurrence of a primary intracranial GCT that responded to salvage therapy with surgical resection, high‐dose chemotherapy (HDCT), and autologous stem cell transplantation (ASCT).

### 1.1 Case

**FIGURE 1:**
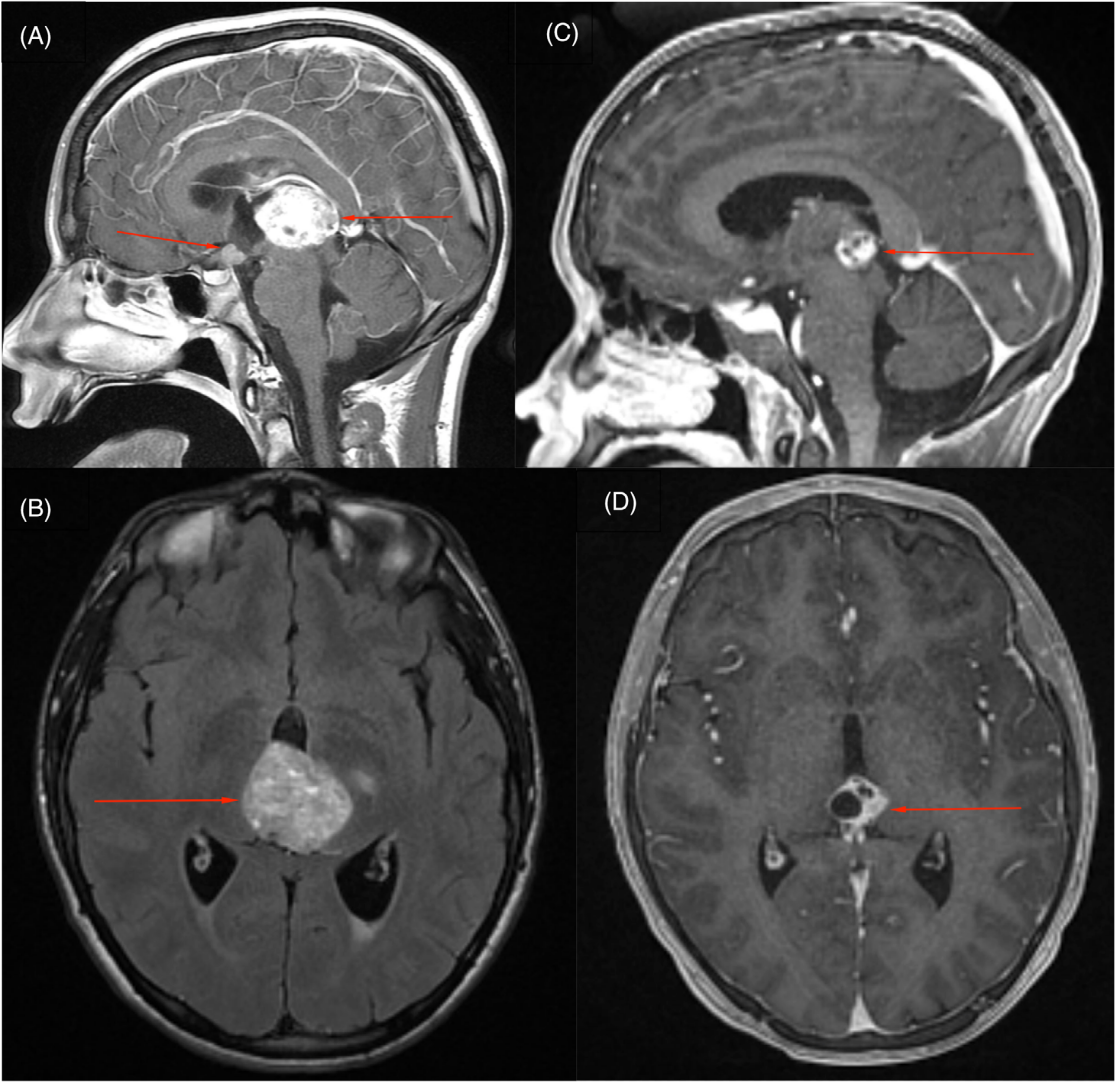
(A,B) Sagittal and axial magnetic resonance images (MRIs) with contrast following presentation in the emergency department demonstrating bifocal suprasellar and pineal tumors. (C,D) Sagittal and axial MRIs with contrast following completion of chemotherapy per Children's Oncology Group ACNS 1123 indicating complete response of the suprasellar tumor and partial response of the pineal tumor

A 15‐year‐old male presented to the emergency department with a 3‐week history of severe morning headaches, nausea, vomiting, and diplopia. Examination revealed bilateral Parinaud syndrome, grade 3 papilledema, and visual acuity of 20/40, and right‐sided cranial nerve VI palsy. Magnetic resonance imaging (MRI) of the brain revealed non‐communicating hydrocephalus, bifocal intracranial tumors, and significant periventricular edema (Figure 1A,B). A 3.7 cm pineal tumor obstructed the aqueduct of Silvius, and a 0.9 cm suprasellar tumor involved the pituitary stalk. Serum tumor markers for alpha‐fetoprotein (AFP) and β‐human chorionic gonadotropin (β‐hCG) measured 678 ng/ml and 40 international units(IU)/L, respectively.

The patient underwent third ventriculoscopy and biopsy of the suprasellar tumor via a right frontal burr hole the day after admission. Cerebrospinal fluid (CSF) levels of AFP and β‐hCG were elevated to 76 ng/ml and 67 IU/L, respectively. The suprasellar tumor was histologically determined to be a nongerminomatous germ cell tumor (NGGCT) of mixed origin. Over the following 6 months, the patient underwent 6 cycles of chemotherapy per the Children's Oncology Group ACNS 1123 protocol, with alternating cycles of carboplatin/etoposide and ifosfamide/etoposide, which were complicated by diabetes insipidus. A repeat MRI revealed complete response of the suprasellar mass and a partial response of the pineal tumor, which became more cystic and measured 1.8 cm (Figure 1C,D). CSF markers were normal (AFP = 2 ng/ml, β‐hCG < 1 IU/L). Due to the persistence of the pineal tumor following chemotherapy, suggesting it to be a mature teratoma, second look surgery was planned, and microscopic en bloc resection was achieved via a supracerebellar infratentorial approach. Pathology revealed mature teratoma without residual immature germ cell components. The patient was neurologically intact after the surgery. Treatment proceeded with 6 weeks of craniospinal irradiation (CSI) with protons to 36 gray‐relative biological effectiveness (Gy_RBE_) followed by a boost to 54 Gy_RBE_ for the surgical bed and original sites of the pineal and suprasellar tumors. A follow‐up MRI 1 month after completion of treatment revealed no discernable CNS disease.

**FIGURE 2:**
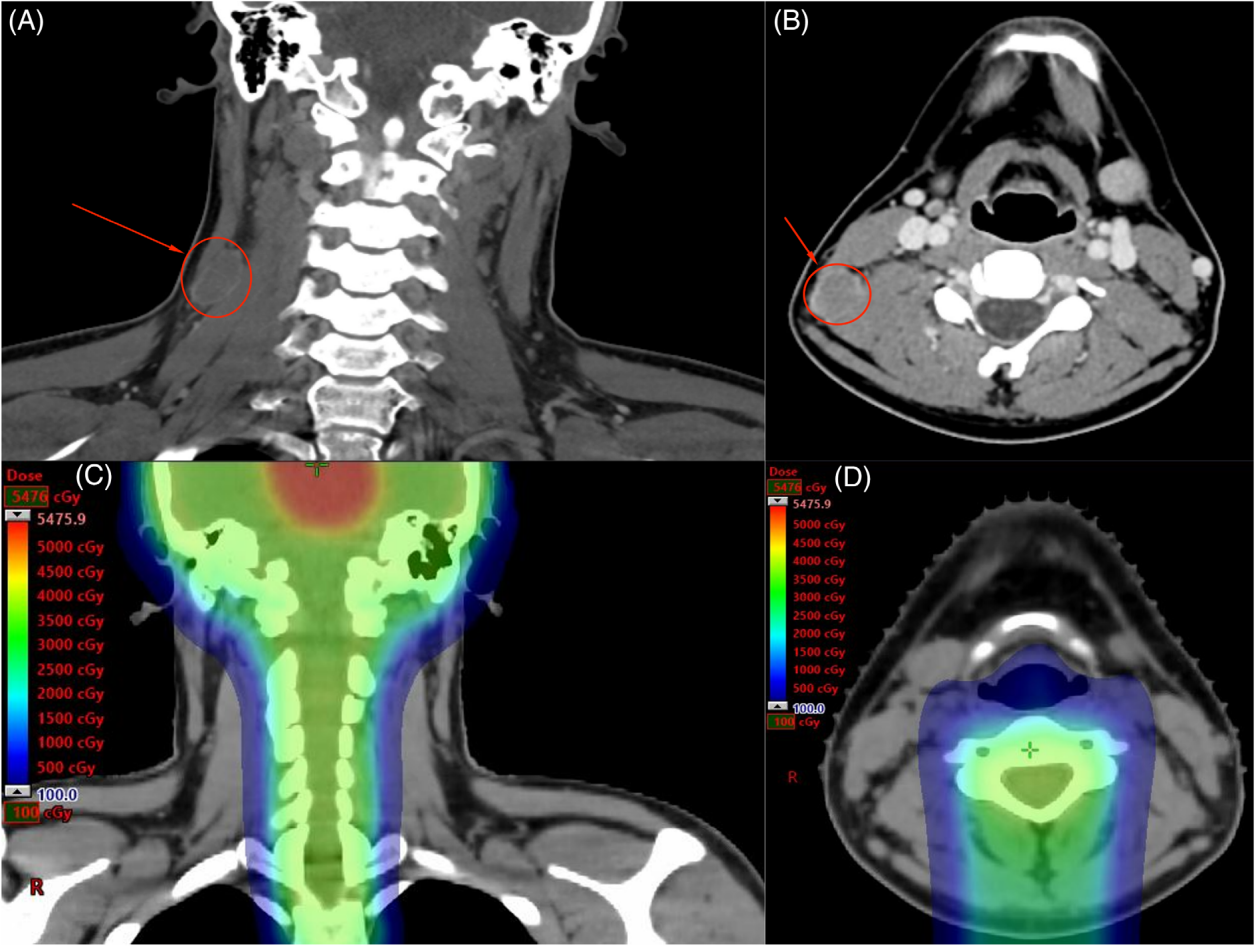
(A,B) After failing response to antibiotics, the enlarged level V right cervical lymph node was imaged with computed tomography. (C,D) This lymph node was located outside the target volume of the patient's prior proton therapy treatment

Nine months following completion of proton therapy, the patient was noted to have an enlarged (2.5 cm) level V right cervical lymph node located outside the prior radiation field. It was initially thought to be reactive. A computed tomography (CT) scan of the node 4 months later showed stable enlargement and a necrotic center (Figure 2A–D). A complete excisional biopsy 1 month later revealed recurrent pure germinoma. A thorough evaluation for additional disease was performed, including MRI of the brain and spine, whole body positron emission tomography (PET)‐CT, CSF cytology, and tumor markers, all of which were negative, indicating an isolated extraneural recurrence. AFP and β‐hCG were undetectable in both the serum and CSF.

Following consultation with our multi‐disciplinary oncology conference, salvage therapy involving HDCT and ASCT followed by focal radiotherapy to the cervical node and adjacent lymphatic levels was planned. The treatment was adopted from work by Adra and colleagues for relapsed GGCTs and utilized two rounds of carboplatin (700 mg/m^2^) and etoposide (705 mg/m^2^), with ASCT and a 21‐day break following each round.^5^ Treatment was complicated by recurrent episodes of diabetes insipidus and hypernatremia, requiring admissions to the pediatric intensive care unit to complete the regimen. He has since been followed closely and currently has no evidence of disease 35 months after diagnosis of recurrence, and 30 months after completing salvage therapy. Currently, the patient is presenting for routine follow‐up with oncology and endocrinology services.

## 2 DISCUSSION

Recurrence of intracranial NGGCTs occurs in 15%–30% of patients despite treatment with multi‐agent chemotherapy, radiation therapy, and surgical resection.^6^, ^7^, ^8^ Like most primary brain tumors, recurrences are almost exclusively in the CNS, most commonly in the site of initial disease, and sometimes involving intra‐ventricular dissemination and drop‐metastases to the spinal cord.^3^, ^9^, ^10^, ^11^, ^12^ Extraneural metastases of intracranial GCTs are rare. Of the few recent case reports, none describe metastasis to lymphoid tissue, and there are no reports of successful salvage therapy with HDCT in this setting.^13^, ^14^, ^15^, ^16^ The optimal salvage treatment for recurrent metastatic germinoma—particularly NGGCTs—is debated, and this rare extraneural presentation introduced additional challenges for the care team in determining a treatment plan, as it was unclear if any consensus treatment guidelines would apply.^17^

The first challenge was to determine areas at risk for harboring micrometastatic disease and whether our patient should be treated for locoregional versus metastatic recurrence. The cervical lymph nodes provide efferent lymphatic drainage from the brain, and while uncommon, isolated metastases of primary brain tumors have been described in the cervical lymph nodes in several older case reports.^18^, ^19^, ^20^, ^21^, ^22^, ^23^, ^24^, ^25^, ^26^ If the disease spread to the lymph node through lymphatic channels directly draining the CNS, this would represent a locoregional recurrence with micrometastatic disease likely confined to the cervical lymph nodes. Alternatively, if the neoplastic cells spread systemically prior to depositing in the lymph node, the entire body would be at risk for harboring micrometastatic disease. Despite the negative metastatic work‐up and the rationale for locoregional recurrence, the possibility of both local and systemic micrometastases could not be ruled out in the setting of such a rare presentation. In the absence of evidence for CNS disease, we decided to approach this patient's disease as if it was a metastatic recurrence of an extracranial GCT, and systemic chemotherapy with or without radiotherapy following complete excisional biopsy was considered the best path forward.

The optimal treatment of relapsed GCTs—both within and outside the CNS—is an area of open debate, and multiple factors contributed to the ultimate choice in chemotherapy regimen. The first concern was the possibility of platinum refractory disease in this patient. Although platinum refractory disease is defined as recurrence during or within 4 weeks of completing initial chemotherapy, the relatively short interval between the completion of initial therapy and recurrence (9 months) was concerning for this element in our patient's disease and prompted our favoring a HDCT regimen followed by ASCT over a conventional‐dose platinum‐based salvage chemotherapy regimen.^27^ However, the superiority of HDCT in the setting of recurrent metastatic seminoma is debated and is currently being investigated in the international prospective phase III TIGER trial (NCT02375204).^28^, ^29^ Second, despite the relatively favorable pathology of recurrence as pure germinoma, there remained a concern for possible micrometastases with nongerminomatous components too small to present with positive serum markers. After consulting with additional outside experts in the treatment of germ cell tumors, the regimen of Adra and colleagues was chosen because their published experience involving 364 patients with recurrent metastatic GGCTs resulted in an estimated 60% 2‐year progression‐free survival and 66% overall survival.^5^ This regimen was found to be efficacious for treating both seminomatous and nonseminomatous metastases, as well as disease in the CNS.

For this young, otherwise healthy patient with potentially platinum refractory disease, HDCT and ASCT were viewed as the best option for achieving long‐term disease control. Had ASCT not been an option for this patient—for medical reasons or due to feasibility or availability—nonmyeloablative regimens used in the treatment of intracranial and extracranial GCTs such as GEMPOX (gemcitabine, paclitaxel, and oxaliplatin), JEB (carboplatin, etoposide, and bleomycin), or PVB (cisplatin, vinblastine, and bleomycin) would have been viable alternatives.^30^, ^31^ However, with less intensive chemotherapy regimens, a greater emphasis would likely have been placed on loco‐regional control with radiotherapy. For instance, in the setting of recurrent CNS disease, these lighter chemotherapy regimens (GEMPOX, specifically) must be followed with myeloablative chemotherapy and radiation to obtain adequate local control, as was recently documented in the final report of GCT‐66.^32^ Additionally, the presence of targetable mutations could have also directed therapy; however, these are unusual in germ cell tumors, and none were revealed upon DNA sequencing of the recurrence after biopsy.

The role of radiotherapy in this situation is unknown. If we could be confident that this relapse represented locoregional recurrence of only the germinomatous component of the GCT in the first echelon draining lymph nodes of the CNS, the analogous situation for a testicular GGCT would call for radiotherapy alone to the draining lymph nodes.^33^ However, we could not exclude the possibility of more widely metastatic microscopic disease within and outside of the CNS. With regard to possible CNS disease, CSI with concurrent chemotherapy is standard of care in the treatment of NGGCTs with CNS metastases; however, repeat CSI would carry unbearable toxicities for our patient.^17^, ^34^ We also considered recommending radiotherapy to the cervical lymph nodes due to concerns over the possibility of micrometastatic nongermanomatous disease, since radiotherapy is an important part of first‐line therapy for NGGCT in the CNS and may be important in the salvage setting as well.^17^ Multiple treatment volumes were considered, including the cervical node site, ipsilateral neck, and bilateral neck. However, due to the significant toxicity potential of the chosen chemotherapy regimen and the additional toxicity expected from more extensive radiotherapy, the care team recommended focal adjuvant radiotherapy to the site of the involved cervical node and adjacent lymphatic levels. It was not clear if this additional treatment was necessary after resection and intensive salvage chemotherapy, but radiotherapy was recommended given the uncertainties and the difficulty of a second salvage, should the disease recur a second time. Ultimately, the patient declined consolidative radiotherapy and currently has no signs of recurrence 30 months after completion of salvage therapy.

## CONFLICT OF INTEREST

The authors have stated explicitly that there are no conflicts of interest in connection with this article.

## AUTHOR CONTRIBUTIONS

All authors had full access to the data in the study and take responsibility for the integrity of the data and the accuracy of the data analysis. *Writing ‐ Original Draft*, J.H.; *Conceptualization*, C.D., J.M.W., L.C., A.E.S., M.D., R.V, K.K.; *Writing ‐ Review & Editing*, R.V., K.K.

## ETHICAL STATEMENT

The patient provided consent to publish this report.

## Data Availability

Data sharing is not applicable to this article as no new data were created or analyzed in this study.
